# Phylogenetic and Molecular Characterization of H9N2 Influenza Isolates from Chickens in Northern China from 2007–2009

**DOI:** 10.1371/journal.pone.0013063

**Published:** 2010-09-29

**Authors:** Jianmin Bi, Guangcun Deng, Jun Dong, Fuli Kong, Xuezhu Li, Qiang Xu, Miaojie Zhang, Lihong Zhao, Jian Qiao

**Affiliations:** Department of Pathophysiology, College of Veterinary Medicine, China Agricultural University, Beijing, People's Republic of China; University of Georgia, United States of America

## Abstract

The repeated transmission to pigs and humans, and the long-term endemicity in terrestrial poultry of H9N2 viruses in China lend urgency to the study of their ecology and pathogenicity. In the present paper, we reported an H9N2 virus sublineage isolated from chickens in northern China from 2007 to 2009 has high lethality for mice. Phylogenetic analysis of the full genome indicated that six representative H9N2 isolates shared high homology to each other, and they clustered in the same sublineage with other H9N2 viruses isolated recently in northern China. The isolates were double-reassortant viruses containing M genes similar to A/Quail/Hong Kong/G1/97 (H9N2) and the other seven gene segments from A/Chicken/Shanghai/F/98 (H9N2). These six isolates were capable of replicating in the lungs of infected chickens without producing observable clinical signs of disease or death. However, they were highly lethal to mice with mortality rates as high as 100% (14/14) without prior adaptation. The affected mice exhibited severe respiratory syndromes and diffuse lung injury. The H9N2 viruses could be detected in multiple organs of the infected mice, including hearts, livers, spleens, lungs and kidneys. Our findings demonstrated that H9N2 viruses isolated from the chickens in northern China have established a stable sublineage with enhanced pathogenicity to mice, suggesting that urgent attention will need to be paid to the transmission of H9N2 viruses from chickens to mammals.

## Introduction

Epidemiological studies have revealed that H9N2 viruses exist on all continents except Antarctica. In North America, H9N2 viruses have been found mainly in shorebirds and wild ducks with no evidence of permanent lineages of these viruses established in terrestrial poultry [1], [2], since the first H9N2 virus was isolated from turkeys in 1966 [3]. In contrast, H9N2 viruses are endemic in different types of terrestrial poultry in multiple countries on the Eurasian continent. In Europe, H9N2 viruses have been isolated from domestic poultry, including turkeys, chickens, pheasants, and domestic ducks, between 1995 and 1997 [4]. In Asia, H9N2 viruses were detected only in apparently healthy ducks from live poultry markets in Hong Kong from 1975 to 1985 [5]. The H9N2 viruses were first isolated from terrestrial birds (quails) in Hong Kong in 1988, and they became prevalent in live poultry markets during 2001–2003 [6]–[8]. In the mainland of China, H9N2 viruses were first isolated from diseased chickens in Guangdong province in 1994, and have since spread to domestic poultry in the other provinces [8]–[17].

Recent studies have suggested that H9N2 viruses were able to occasionally transmit from terrestrial poultry to mammals, including swine and humans. Since 1988, repeated H9N2 viral infections have been reported in swine with apparent clinical disease in China [18]–[20]. Mild respiratory disease in humans was also reported in Hong Kong and the mainland of China in 1999, and again in Hong Kong in 2003 [21]–[23]. Genetic analysis demonstrated that the H9N2 viruses isolated from human likely originated directly from avian origin [21], [23], [24], providing the preliminary support that the avian H9N2 viruses were potentially infectious for humans. The infectivity of avian H9N2 viruses for humans was further supported by in vitro trials which indicated some of the poultry H9N2 isolates contain Leucine (Leu)-226 in the receptor-binding site (RBS) of hemagglutinin (HA), which is typical of human H2 and H3 viruses [25], [26]. Additionally, previous studies demonstrated that Leu226-containing H9N2 viruses exhibited human virus-like receptor specificity, i.e. they bind efficiently to sugar moieties terminated with α2,6 sialic acid (SAα2,6) [8], [25], [26]. Further evidence for host range expansion of the H9N2 viruses with Leu226 is the observation that these strains can replicate in ferrets and be transmitted between individuals by direct contacts [27]. Collectively, these studies highlight the necessity for more comprehensive surveillance and further evaluation of H9N2 viruses.

Although influenza pathogenesis in mice is not fully consistent with that in humans, mouse experiments have been widely used to better understand the potential of influenza viruses to cause disease in humans. In addition, mice have been used as an animal model to reflect the severity and outcome of disease in humans infected by Eurasian-lineage H5N1 high pathogenic avian influenza (HPAI) virus [28]–[30]. Comparatively, very few experimental trials have examined influenza viruses of the H9 subtype in mice. Previous studies indicated that the H9N2 viruses isolated in China were heterogeneous in their pathogenicity for mice; some isolates were pathogenic and replicated systemically with high viral titers [8], [12], [13], [31], while others were less pathogenic and replicated only in respiratory organs [12], [13], [32]. The prevalence of avian H9N2 viruses throughout Asia, along with their demonstrated ability to infect mammals, puts them high on the list of influenza viruses with pandemic potential for humans, and emphasizes the importance of continued surveillance, isolation, and characterization of H9N2 viruses present in poultry. In this study, we reported an H9N2 virus sublineage isolated from chickens in northern China with high lethality to mice. Our data demonstrated that the full genome of six representative isolates shared high homology to each other, and clustered in the same lineage with other H9N2 viruses isolated recently in northern China. These viruses were able to replicate efficiently in mouse lungs, and could cause 100% mortality without prior adaptation [31]. These findings indicated that H9N2 viruses isolated from northern China have expanded their host ranges, which might favor the emergence of H9N2 viruses with pandemic potential in humans.

## Results

### Background information and prevalence of H9N2 viruses in chickens

During the winter of 2007 to the spring of 2008, two unusual H9N2 viruses were isolated, A/Chicken/Hebei/4/08(Ck/HB/4/08) [31] and A/Chicken/Hebei/A/07(Ck/HB/A/07), from chickens with severely reduced egg production in northern China. Previous experiments indicated that both viruses were highly lethal for mice, producing 100% mortality (n = 10) without prior adaptation after exposure to 10^7^ of fifty percent egg infectious dose (EID_50_) of either virus [31]. Considering the higher than expected mortality rates in mice, we expanded the H9N2 virus surveillance to a wider region covering 250 thousand square kilometers to the north of the Yellow River, including Hebei and Henan province in northern China. This area included a population of 100 million laying hens. From November 2007 to March 2009, 106 chicken samples (lungs and oviducts) were collected from 45 poultry farms in different regions experiencing severe drops in egg production. Ninety-eight strains of H9N2 viruses were isolated from the 106 chicken samples (isolation rate, 92.5%). Of these isolates, 72 were from lung samples. Based on the date and site of sampling, 23 representative virus isolates were selected to test for lethality in mice. The results showed that these 23 viruses were all highly lethal to mice, producing 90%–100% mortality (n = 10) without prior adaptation after exposure to 10^7^ EID_50_ of each virus (data not shown). Subsequently, 6 of the 23 isolates from different sampling dates and sites were chosen as representative isolates for further genetic and biological characterization (Table 1).

**Table 1:** Background information of avian influenza H9N2 viruses examined in this study.

Virus	Abbreviation	Date of isolation	MID_50_ a	MLD_50_ a	Lethality^b^in chickens	GenBankaccession No.
A/Chicken/Hebei/A/07	Ck/HB/A/07	Nov 2007	2.0	6.0	Non	GQ202056–GQ202063
A/Chicken/Henan/1.2/08	Ck/HN/1.2/08	Jan 2008	1.2	5.7	Non	FJ534538–FJ534545
A/Chicken//Hebei/4/08	Ck//HB/4/08	Jan 2008	1.7	6.0	Non	FJ499463–FJ499470
A/Chicken/Hebei/7/08	Ck/HB/7/08	Mar 2008	2.5	6.5	Non	FJ534546, FJ534547,GQ202050–GQ202055
A/Chicken/Hebei/C4/08	Ck/HB/C4/08	Apr 2008	1.7	6.3	Non	FJ534548, FJ534549,GQ202044–GQ202049
A/Chicken/Hebei/Y2/09	Ck/HB/Y2/09	Mar 2009	2.1	6.4	Non	GQ202036–GQ202043

a Expressed as the log_10_ EID_50_ required to give 1 MID_50_ or 1 MLD_50_.

b Lethality was determined by the mortality in 4-wk-old SPF chickens with intranasal infection with each of six H9N2 viruses at a dose 10^7^ EID_50_. “Non” means no death of chickens over 21-day observation period.

### Phylogenetic analysis

To determine the genetic and evolutionary characterization of H9N2 viruses isolated from chickens in northern China, all eight of gene segments from each of six isolates were sequenced, and phylogenetically analyzed. The nucleotide sequences were compared with the sequences of other representative viruses of swine and human H9N2 viruses and Eurasian-lineage H5N1 viruses obtained from GenBank [8], [10], [12]–[14], [18].

**Figure 1:**
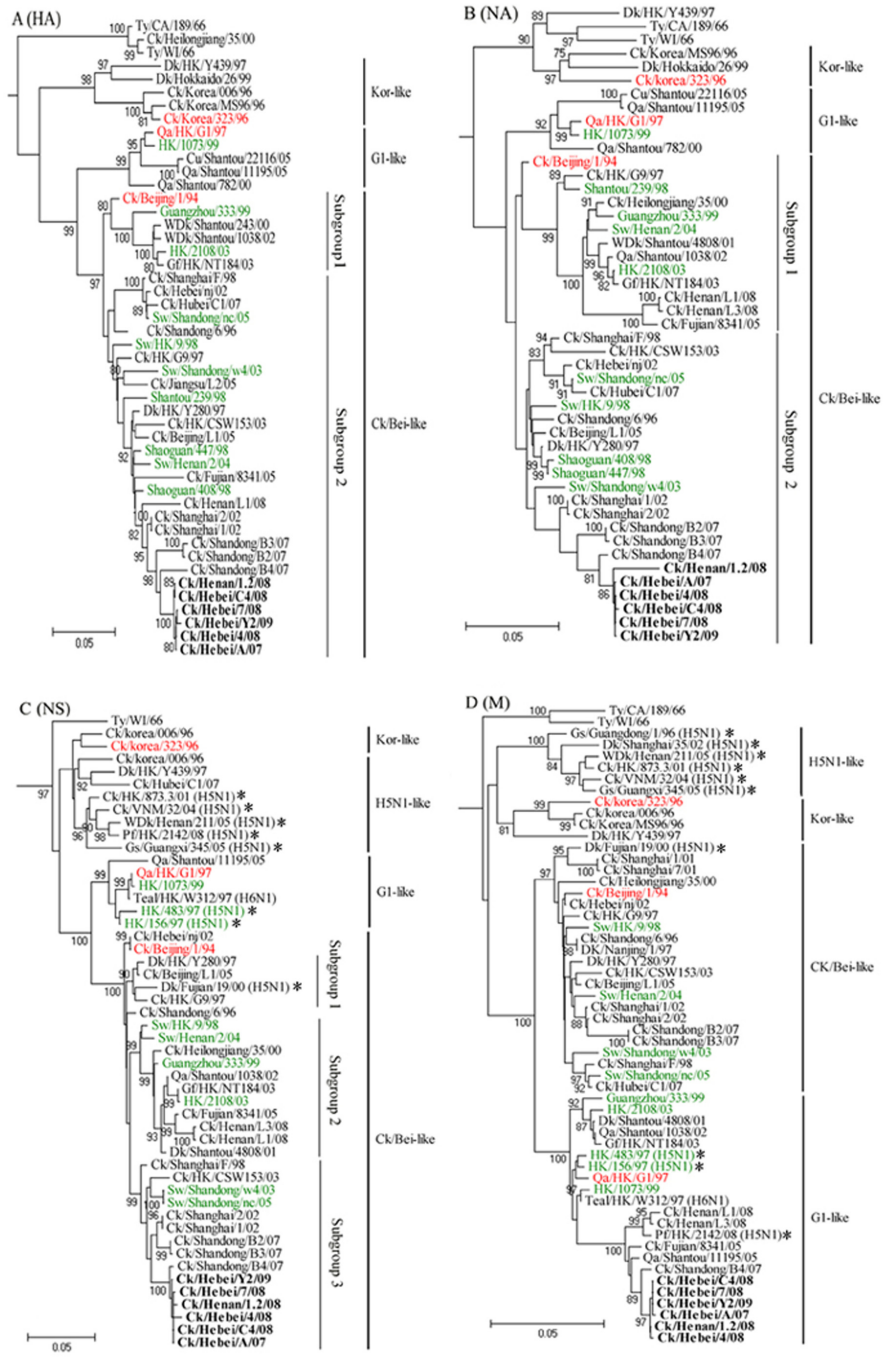
Phylogenetic trees for HA (*A*), NA (*B*), M (*C*) and NS (*D*) genes of the H9N2 influenza viruses analyzed. Trees were generated by the neighbor-joining method with the MEGA program (version 4.1). Nucleotides 80–1090 (1011 bp, HA), 20–1420 (1401 bp, NA), 26–984 (959 bp, M) and 41–859 (819 bp, NS) were used for phylogenetic analysis. The HA phylogenetic tree is rooted to A/Duck/Alberta/60/76(H12N5), and the trees of NA, M, and NS are rooted to A/Equine/Praque/1/56(H7N7). The length of the horizontal lines is proportional to the minimum number of nucleotide differences required to join nodes. Vertical lines are for spacing and labeling. Viruses characterized in this study are highlighted in bold, representative H9N2 viruses are in red, viruses isolated from swine and human are in green, and H5N1 influenza viruses are highlighted with asterisk. Abbreviations: Ck, chicken; Dk, duck; Sw, swine; Gf, guinea fowl; Pg, pigeon; Gs, goose; Qa, quail; Ty, turkey; Pf, peregrine falcon; WDk, wild duck; Cu, chukar; HK, Hong Kong; WI, Wisconsin, Bei, Beijing; CA, California; VNM; Viet Nam.

As reported in previous genetic studies of HA and NA genes [10]–[14], H9N2 virus infections in poultry were mainly caused by three distinct lineages of H9N2 viruses. These viruses include Ck/Bei-like viruses represented by A/Chicken/Beijing/1/94 (Ck/BJ/1/94), G1-like viruses represented by A/Quail/Hong Kong/G1/97 (Qa/HK/G1/97), and Y439-like or Korean-like viruses represented by A/Duck/Hong Kong/Y439/97. In the current study, phylogenetic analysis of HA and NA genes showed that all six isolates shared 99.6%–100% and 97.3%–99.9% identity to each other, respectively, and all belonged to the Ck/Bei-like lineage (Fig. 1A and 1B). Since the first isolation of Ck/Bei-like viruses from southern China in the mid-1990s, these viruses have evolved gradually and conservatively. Included in this lineage are most of the H9N2 viruses isolated from poultry (chicken, duck and pigeon). The Ck/Bei-like viruses have reportedly been prevalent in China in recent years [8], [13], [14], [16]. Of this lineage, two subgroups have been recognized; subgroup 1 represented by A/Quail/Shantou/1038/02 and subgroup 2 represented by A/Duck/Hong Kong/Y280/97 or A/Chicken/Shanghai/F/98 (Ck/SH/F/98) [12], [14], [15], [17]. In the present study, the HA and NA genes of the six viruses fell into the subgroup 2 (Fig. 1A and 1B).

The NS genes of recent H9N2 isolates from China clustered within the Ck/Bei-like lineage that could be divided into three subgroups (Fig. 1C). The NS genes of our six isolates, sharing 97.9%–100% identity to each other, all fell into subgroup 3 represented by Ck/SH/F/98. The M genes of the six isolates shared 99.6%–100% homology with each other and all belong to the G1-like lineage (Fig. 1D). Historically, G1-like M genes of H9N2 virus have been rarely detected in chicken flocks in China [12], [14], [33]. It is noteworthy that human H9N2 influenza viruses isolated in Hong Kong and Mainland China, H9N2 viruses that were lethal in mice, and some of the human H5N1 viruses all have G1-like M genes (Fig. 1D).

**Figure 2:**
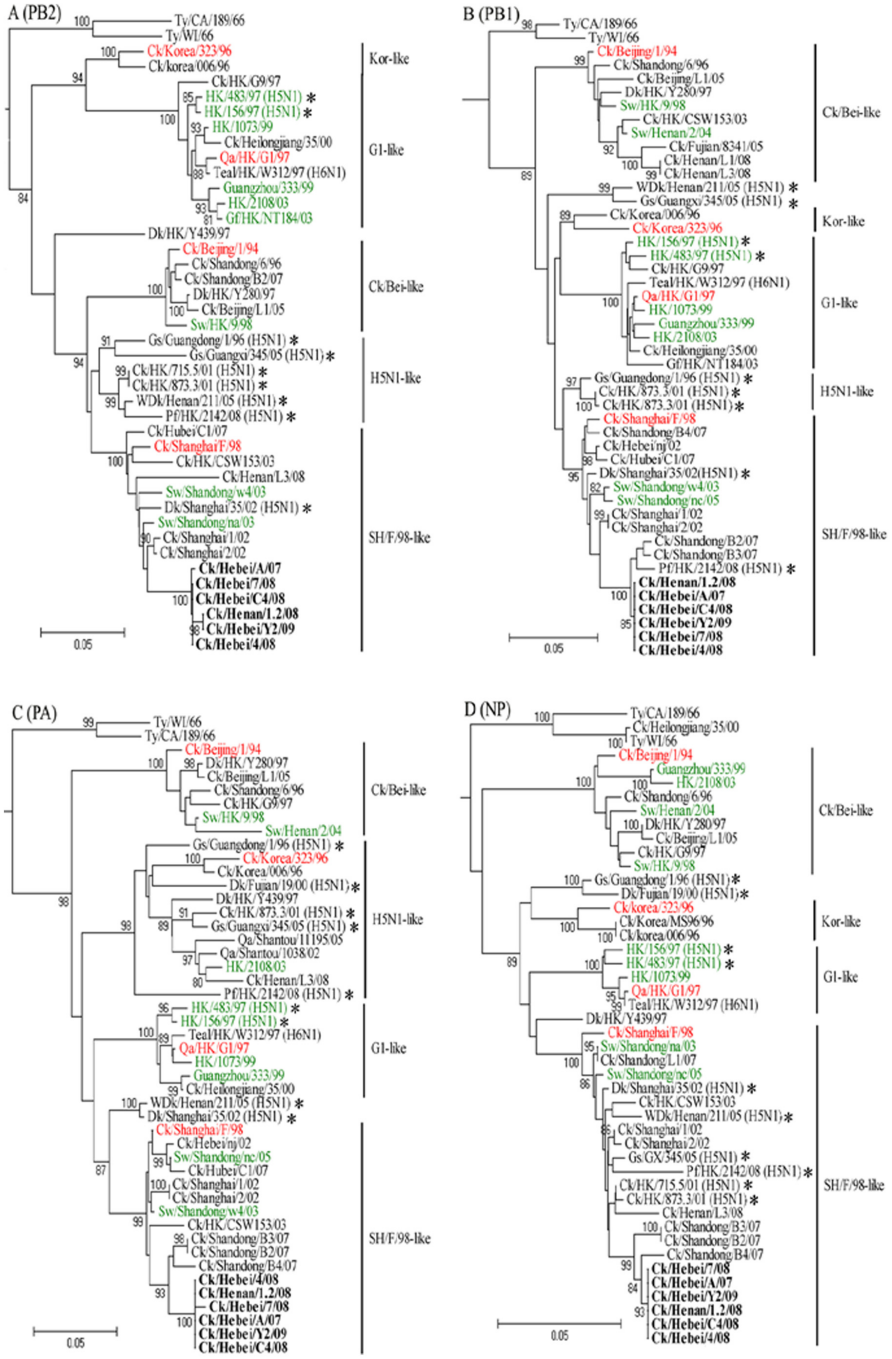
Phylogenetic trees for PB2 (*A*), PB1 (*B*), PA (*C*) and NP (*D*) genes of the H9N2 influenza viruses analyzed. Trees were generated on the basis of the following gene sequences: nucleotides 28–2307 (2279 bp, PB2), 40–1473 (1434 bp, PB1), 25–2175 (2151 bp, PA) and 46–1542 (1497 bp, NP). The PB2 and NP trees are rooted to A/Equine/Praque/1/56(H7N7), and the PB1 and PA trees to A/Equine/London/1416/73(H7N7). The other information was described in Fig. 1.

Phylogenetic analysis of the ribonucleoprotein (RNP) complex genes (PB2, PB1, PA, and NP genes) revealed that the topologies of these four gene trees were very similar (Fig. 2A–2D). The four RNP genes of the six H9N2 viruses shared high identity (more than 99%) to each other, and clustered into the Ck/SH/F/98-like lineage. We noted that the RNP complex genes of a few Eurasian-lineage H5N1 HPAI viruses (highlighted with asterisks in Fig. 2) isolated in China after year 2001 shared high identity to those of our isolates, and also clustered into the Ck/SH/F/98-like lineage. It is interesting to note that the PB1, NP and M genes of the H5N1 HPAI virus A/Peregrine falcon/Hong Kong/2142/08 (highlighted with asterisks in Fig. 2) clustered into the same sublineage with our six H9N2 isolates.

Taken together, the phylogenetic analysis clearly indicated that the six H9N2 viruses tested here possessed G1-like M gene and Ck/SH/F/98-like HA, NA, NS and RNP genes, and formed an independent double-reassortant sublineage with other H9N2 viruses isolated from northern China in recent years (Fig. 1 and Fig. 2).

### Pathogenicity of the H9N2 isolates in chickens

**Figure 3:**
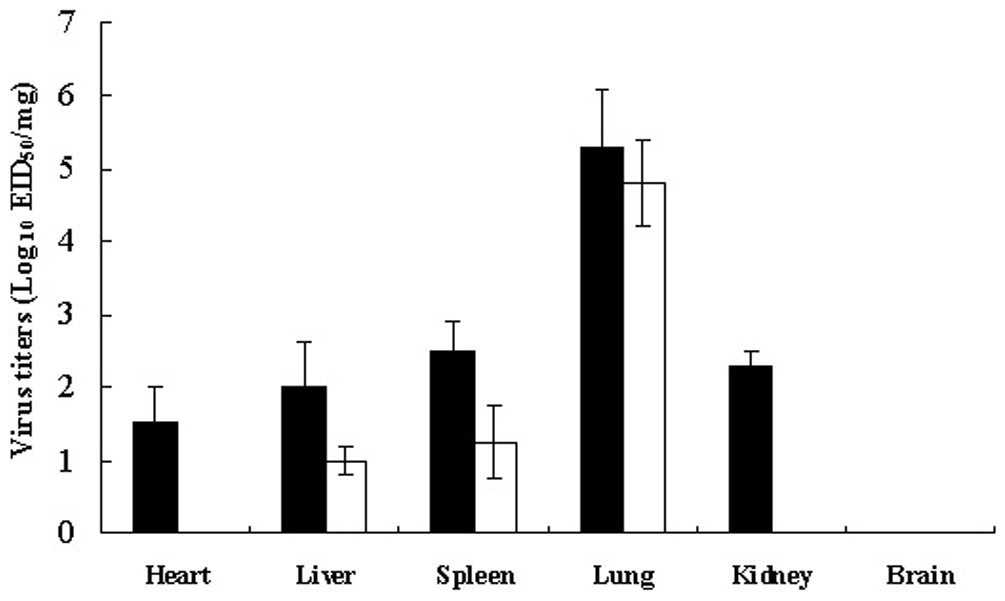
Replication of H9N2 viruses in tissues of the infected mice and chickens. Mice and chickens were inoculated i.n. with Ck/HB/4/08 virus at a dose of 10^6^ EID_50_ (mice), or 10^7^ EID_50_ (chickens). Tissues were collected on 5 days p.i., and the virus was titrated in embryonated eggs from initial dilutions of 1∶10. Mean viral titers based on three mice or chickens per group are expressed as log_10_ EID_50_ per 10 milligrams wet tissues ± SD. Virus is below the detectable level at dilution of ≥10^−1^. *Open bars*, chicken group; *solid bars*, mouse group.

Experimental studies in chickens showed that none of the six viruses induced observable clinical disease or death in specific-pathogen-free (SPF) chickens over the 21-day observation period, except for a slight decline in feed intake and weight gain compared with control birds (data not shown). To evaluate the replication of H9N2 viruses in chickens, tissues were collected for virus titration. The tropism of each tested H9N2 isolate was similar among the six evaluated isolates (Fig. 3, only showing Ck/HB/4/08 virus). All H9N2 virus isolates preferentially replicated in the lungs of the chicken (Table 2). On 5 days post-inoculation (p.i.), viral titers for the different H9N2 isolates ranged from 10^3.8^ to 10^4.8^ EID_50_ per 10 milligrams of wet lung tissues and less than 10^1.3^ EID_50_ per 10 milligrams of the wet tissues from livers and spleens. No virus was detected in hearts, kidneys, and brains of the infected chickens.

**Table 2:** Replication of H9N2 viruses in lungs of mice and chickens.

Viruses	Virus titers a							
	3 day p.i.		5 day p.i.		7 day p.i.		14 day p.i.	
	Chicken	Mice	Chicken	Mice	Chicken	Mice	Chicken	Mice
Ck/HB/A/07	3.8 (0.4)	4.9 (0.4)	4.8 (0.3)	5.9 (0.6)	1.9 (0.3)	4.3 (0.8)	<^b^	0.3 (0.1)
Ck/HN/1.2/08	3.9 (0.1)	4.5 (0.2)	4.7 (0.6)	5.7 (0.4)	1.7 (0.2)	4.2 (1.1)	<	<
Ck//HB/4/08	3.8 (0.2)	5.0 (0.1)	4.8 (0.5)	5.3 (1.2)	1.6 (0.2)	4.4 (0.3)	<	0.5 (0.1)
Ck/HB/7/08	3.7 (0.1)	3.8 (0.5)	4.6 (0.4)	5.4 (0.5)	1.0 (0.5)	3.7 (0.4)	<	<
Ck/HB/C4/08	3.0 (0.8)	4.4 (0.3)	4.5 (0.2)	5.4 (0.3)	1.4 (0.4)	3.5 (0.3)	<	<
Ck/HB/Y2/09	3.3 (0.5)	4.1 (0.4)	3.8 (0.4)	5.1 (0.5)	1.3 (0.5)	3.7 (0.6)	<	<

a Mice and chickens (n = 3) were infected i.n. with each of six H9N2 viruses at a dose of 10^6^ EID_50_ (mice), or 10^7^ EID_50_ (chickens). Clarified homogenates of lungs were titrated for virus infectivity in eggs from initial dilutions of 1∶10. Virus endpoint titers were expressed as mean log_10_ EID_50_ per 10 milligram wet tissues ± standard deviation (SD).

b <, no virus detectable at dilution of ≥10^−1^.

### Pathogenicity of the H9N2 isolates in mice

Generally, the host range of avian influenza viruses is restricted in avian species, including wild birds and domestic poultry [4], [34], but some H9N2 isolates have been reported to have the capacity to infect mammals, including mice, pigs, and humans [8], [12], [13], [18]–[23], [31], [32]. To observe the potential pathogenicity of the six H9N2 isolates in mammals, experimental studies in mice were conducted.

**Figure 4:**
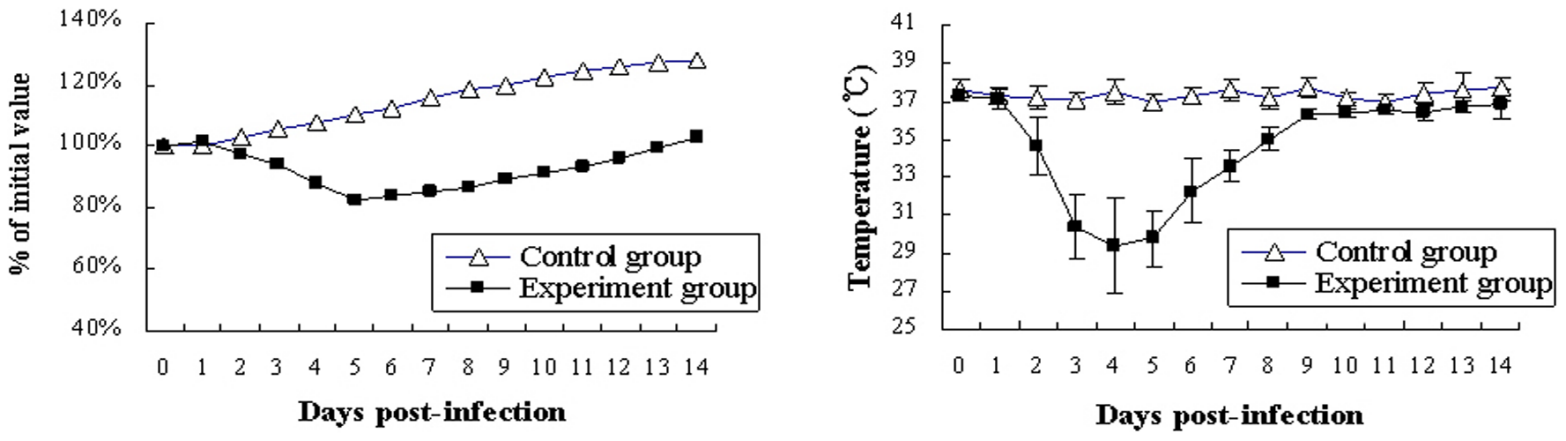
Body weight and temperature of mice after H9N2 viral infection. Mice were inoculated i.n. with 10^6^ EID_50_ of Ck/HB/4/08 H9N2 viruses. Body weight (*A*) and temperature (*B*) were monitored daily for a 14-day observation period. Body weight was expressed as a percentage of the initial value. Data represent the mean of at least three mice of each group.

Most mice (37/42) presented with a relatively acute clinical syndrome following exposure to each isolate at doses of 10^7^, 10^6^ or 10^5^ EID_50_ per 100 µl (single bird inoculation dose). The onset and duration of clinical signs induced by representative H9N2 isolate Ck/HB/4/08 at a dose of 10^6^ EID_50_ were as follows. About 35% of mice (5/14) showed inactivity, altered gait, ruffled fur, inappetence, and weight loss on day 2 p.i. On days 3 to 6 p.i., more than 70% of mice (10/14) exhibited severe inappetence, emaciation and weight loss, and visible signs of labored respirations and respiratory distress. The onset of inappetence and inactivity was correlated with gradual loss of body weight till the death of animal (Fig. 4). The body temperature also declined over the course of infection until death (Fig. 4). Mice that survived began to recover clinically starting at 7 day p.i.

**Figure 5:**
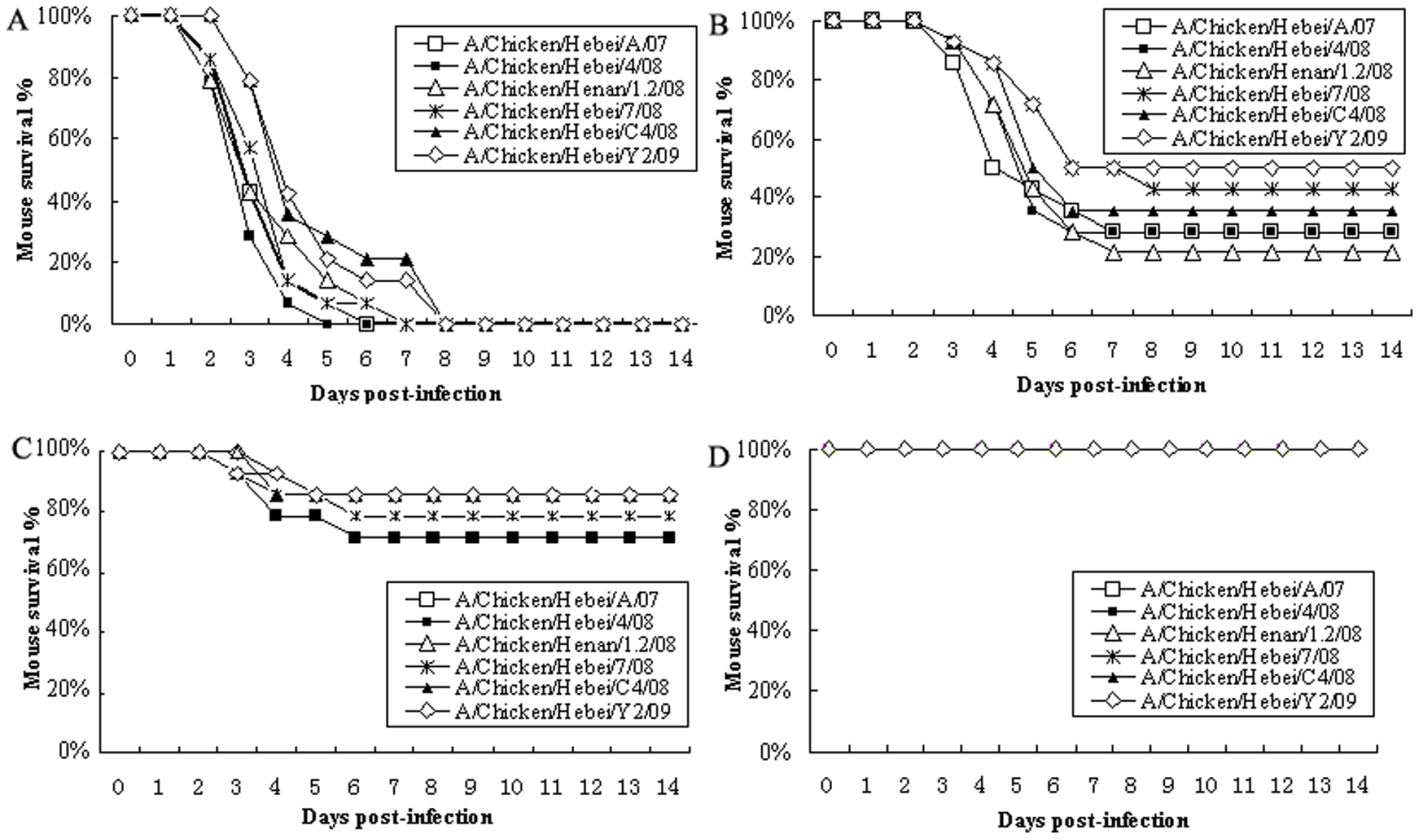
Survival percentage of mice after H9N2 viral infection. Groups of mice (n = 14) were inoculated i.n. with each of six H9N2 viruses at a dose of 10^7^ EID_50_ (*A*), 10^6^ EID_50_ (*B*), 10^5^ EID_50_ (*C*) or 10^4^ EID_50_ (*D*), respectively. Percent survival was observed daily for 14 days.

Mortality rates of mice were shown in Fig. 5 following inoculation of each isolate at doses of 10^7^, 10^6^, 10^5^ or 10^4^ EID_50_. All of the mice exposed to a high dose (10^7^ EID_50_) of each isolate succumbed to respiratory distress on days 2 to 8 p.i. Mortalities for the six virus isolates ranged from 50% to 85.7% on 3 to 7 days p.i., when mice were exposed to 10^6^ EID_50_. After exposure to 10^5^ EID_50_ of each of the six isolates, an average of 19.3% (range from 14.3% to 29.6%) of the infected mice died. None of the mice died after exposure to 10^4^ EID_50_ of any of the six isolates. None of the negative control mice exhibited clinical disease or death. The 50% mouse infection dose (MID_50_) and 50% mouse lethal dose (MLD_50_) of the 6 isolates are listed in Table 1.

Grossly, mice infected with each isolate at a dose of 10^7^, 10^6^ or 10^5^ EID_50_, had markedly edematous lungs with profuse areas of hemorrhage. Infected mice displayed a consistent histopathological lesions consisting of diffuse pneumonia and alveolar damage, including interstitial and alveolar edema, hemorrhage, and severe bronchiolitis/peribronchiolitis (data not shown). Above pathological changes were consistent with our previous report [31].

Viral replication was evaluated in various tissues of infected mice at 5 days p.i. with each of six H9N2 isolates at doses of 10^7^, 10^6^ or 10^5^ EID_50_. Each of six viruses replicated efficiently to high titers in the lungs from mice exposed to each dose (Table 3). Low viral titers were also detected from hearts, spleens, livers and kidneys of the mice after exposure to 10^7^, 10^6^ or 10^5^ EID_50_ of any of the six isolates (Table 3). More importantly, the H9N2 viruses were detected in the brains of mice (1/3) infected with Ck/HN/1.2/08 and Ck/HB/7/08 H9N2 viruses (Table 3). These results indicated that these six H9N2 isolates were all able to replicate systemically in mice, which could explain the high lethality observed in the high dose (10^7^ EID_50_ and 10^6^ EID_50_) exposure groups. In addition, all six H9N2 isolates were able to replicate more efficiently in mouse lungs than in chicken lungs (Table 2).

**Table 3:** Replication of H9N2 viruses in tissues of mice.

Virus	Dose (EID_50_)	Mean virus titers^a^					
		Heart	Liver	Spleen	Lung	Kidney	Brain
Ck/HB/A/07	10^5^	0.3 (1/3)^b^	1.0 (2/3)	1.3 (2/3)	4.1 (3/3)	0.5 (1/3)	<^c^ (0/3)
	10^6^	0.9 (2/3)	1.3 (3/3)	1.5 (2/3)	5.9 (3/3)	1.0 (2/3)	< (0/3)
	10^7^	2.0 (3/3)	2.0 (3/3)	2.4 (3/3)	6.3 (3/3)	1.4 (3/3)	< (0/3)
Ck/HN/1.2/08	10^5^	0.3 (1/3)	0.6 (1/3)	0.9 (2/3)	4.0 (3/3)	0.5 (1/3)	< (0/3)
	10^6^	0.8 (2/3)	1.4 (3/3)	1.9 (3/3)	5.7 (3/3)	1.1 (2/3)	< (0/3)
	10^7^	1.7 (3/3)	1.9 (3/3)	2.2 (3/3)	6.0 (3/3)	2.1 (3/3)	0.3 (1/3)
Ck//HB/4/08	10^5^	0.6 (2/3)	0.8 (2/3)	1.1 (2/3)	3.8 (3/3)	0.8 (2/3)	< (0/3)
	10^6^	1.5 (3/3)	2.0 (3/3)	2.4 (3/3)	5.3 (3/3)	2.2 (3/3)	< (0/3)
	10^7^	1.4 (3/3)	2.1 (3/3)	2.9 (3/3)	6.1 (3/3)	2.6 (3/3)	< (0/3)
Ck/HB/7/08	10^5^	0.6 (1/3)	0.9 (2/3)	1.0 (2/3)	3.8 (3/3)	0.7 (1/3)	< (0/3)
	10^6^	1.1 (3/3)	1.0 (2/3)	1.8 (3/3)	5.4 (3/3)	1.4 (2/3)	0.3 (1/3)
	10^7^	1.5 (3/3)	1.9 (3/3)	2.3 (3/3)	6.0 (3/3)	2.5 (3/3)	0.3 (1/3)
Ck/HB/C4/08	10^5^	0.6 1/3)	0.7 (1/3)	0.9 (2/3)	3.7 (3/3)	0.6 (1/3)	< (0/3)
	10^6^	1.0 (2/3)	1.2 (2/3)	2.2 (3/3)	5.4 (3/3)	0.8 (1/3)	< (0/3)
	10^7^	1.3 (2/3)	1.8 (2/3)	2.3 (3/3)	6.2 (3/3)	1.6 (2/3)	< (0/3)
Ck/HB/Y2/09	10^5^	< (0/3)	0.6 (1/3)	0.7 (1/3)	3.1 (3/3)	< (0/3)	< (0/3)
	10^6^	0.7 (1/3)	1.3 (2/3)	1.5 (2/3)	5.1 (3/3)	0.8 (1/3)	< (0/3)
	10^7^	1.0 (2/3)	1.4 (2/3)	1.8 (2/3)	5.8 (3/3)	1.7 (2/3)	< (0/3)

a Mice (n = 3) were inoculated i.n., and tissues were taken on days 5 p.i. Clarified homogenates of tissues were titrated for virus infectivity in eggs from initial dilutions of 1∶10. Mean viral titers are expressed as log_10_ EID_50_ per 10 milligram wet tissues.

b Average virus titers for each tissue (the number of positives/total number tested for each virus).

c <, no virus detectable at dilution of ≥10^−1^.

### Molecular Characterization of the H9N2 isolates

To identify the potential determinants for the higher lethality of the H9N2 isolates for mice, the amino acid sequences of the HA, NA and PB2 genes were aligned and compared with those of other representative H9N2 viruses.

Although the virulence of avian influenza viruses is a polygenic property, the amino acid sequence of the connecting peptide of HA has been considered a major determinant in terrestrial poultry [35]–[37]. In this study, all isolates shared an identical amino acid sequence of PARSSR↓G (residues 324–330, numbering according to H3 HA) at the cleavage sites (arrow) between HA1 and HA2 (Table 4), a characteristic of low pathogenic avian influenza virus found in terrestrial poultry [4], [34]. The receptor specificity of the viral HA is believed to be a factor that may limit the generation of human pandemic viruses from avian precursors [38]. The HA of four H9N2 viruses tested in our study had Leu226 at RBS (numbering according to H3 HA), a typical residue found in human H2 and H3 pandemic strains [8], [25], [26], [38]. The other two H9N2 viruses in our study had Glutamine (Glu) at position 226 in HA (Table 4). Changes at potential glycosylation sites (PGSs) of the HA have been thought to be associated with the adaptation of duck viruses to poultry [39]. Seven PGSs were present in the HA of all six isolates, with the N-X-T/S motif (in which X may be any amino acid except Proline), including five PGSs in HA1 (10, 123, 200, 280, and 287, numbering according to H3 HA) and two PGSs in HA2 (474 and 533, numbering according to H3 HA).

**Table 4:** Comparison of amino acid sequences of HA and NA of H9N2 viruses tested in this study

Virus	Residue at RBS^a^ (H3 number)			Connecting peptidesequences (aa)^b^	NA deletion(aa)
	183	190	226		
Ck/HB/A/07	N	V	L	PARSSRG	62–64
Ck/HN/1.2/08	N	A	L	PARSSRG	11–12; 62–64
Ck//HB/4/08	N	V	Q	PARSSRG	62–64
Ck/HB/7/08	N	V	L	PARSSRG	62–64
Ck/HB/C4/08	N	A	L	PARSSRG	62–64
Ck/HB/Y2/09	N	V	Q	PARSSRG	62–64

a RBS, receptor binding site.

b Amino acid sequence of residues 324–330 (numbering according to H3 HA) at the cleavage site between HA1 and HA2; aa, amino acid.

It has been postulated that shortening of the NA stalk by deletion of amino acids is characteristic of highly pathogenic avian influenza viruses [40]. Five of six H9N2 isolates examined in this study had the identical deletion of three amino acids (positions 62–64) in the NA stalk region, as previously described [13]. While, the isolate Ck/HN/1.2/08 had another two amino acid deletion (positions 11–12), different from other H9N2 viruses reported previously (Table 4) [12], [13]. Hulse et al. revealed that an additional PGS within the NA protein globular head contributed to the high virulence of H5N1 HPAI viruses in chickens [41]. Analysis of the NA sequences of the six H9N2 isolates revealed the presence of at least seven PGSs. It is worth to note that Ck/HN/1.2/08 possesses nine PGSs.

In addition, the presence of lysine (Lys) at position 627 of the PB2 protein is believed to favor the replication of influenza viruses in mice and thus considered an important host range determinant [42]–[44]. In the present study, all 6 H9N2 viruses have Glu at position 627 of the PB2 proteins.

## Discussion

H9N2 viruses have frequently been detected in terrestrial poultry in China since 1994, and have established several permanent lineages in Asia [10]–[14], [17]. More importantly, H9N2 viruses have occasionally transmitted from domestic poultry to mammals, including pigs and humans [18]–[24]. Recent studies have demonstrated that some H9N2 virus isolates could replicate in mice and ferrets without prior adaptation [8], [12], [13], [27], and that some H9N2 viral strains, closely related to those that crossed to humans and pigs, continue to circulate in China [13], [14]. In addition, a number of recent H9N2 isolates have typical features of human influenza viruses, such as the ability of efficient binding SAα2,6 receptors [8], [25], [26]. These observations suggest that H9N2 viruses have progressively gained replication properties that make them more adapted to mammals and may represent a potential infectious agent to humans. Consequently, H9N2 viruses warrant further study in an effort to understand their pathogenesis in mammals.

In the present study, we reported an H9N2 virus sublineage, isolated from chickens in northern China from 2007 to 2009, with higher than expected lethality in mice. The phylogenetic analysis of the full genomes indicated that the six representative H9N2 viruses possessed G1-like M genes and Ck/SH/F/98-like HA, NA, NS and RNP genes. These viruses comprised an independent double-reassortant sublineage with other H9N2 viruses isolated from northern China in recent years. The similarity scores calculated from alignments supported the phylogenetic results. Although the six H9N2 viruses were isolated from chickens at different times and locations, all eight genes of these isolates shared high nucleotide homology with each other, and fell into the same cluster, suggesting that they were of the same origin. Since Ck/SH/F/98 H9N2 virus, a natural reassortant containing Ck/Bei-like HA, NA, M, NS and the unique four RNP complex genes, was first isolated from diseased chickens in eastern China in 1998 [17], Ck/SH/F/98-like viruses have gradually become prevalent in chicken flocks in China [13], [15]. The data presented in this report suggests that Ck/SH/F/98-like viruses have continued to evolve through reassortment with other H9N2 viruses, and have been replaced by the new reassortant sublineage in chickens in northern China.

The experimental trials in chickens showed that the six H9N2 isolates tested herein were able to replicate in chicken lungs, but did not produce observable clinical disease or death over a 21-day observation period. Consequently, these H9N2 isolates, like other H9N2 viruses, are of low pathogenicity in chickens.

Finally, these six H9N2 viruses were highly lethal to mice and could cause 100% mortality without prior adaptation after exposure to 10^7^ EID_50_ of virus. Mortality ranged from 50% to 85.7%, when lower infectious doses of virus (10^6^ EID_50_) were used. Severe respiratory syndromes associated with diffuse lung injury, severe pneumonia and alveolar damage were observed in clinically affected mice. At 5 days after exposure to 10^7^, 10^6^ or 10^5^ EID_50_ of each H9N2 isolate, viruses were detected in multiple organs of the mice, including hearts, livers, spleens, lungs and kidneys. Of these organs, the most efficient replication was in lungs. These findings suggested that H9N2 viruses have established a stable sublineage with enhanced pathogenicity to mice in chickens in northern China.

Generally, the host range of avian influenza viruses is restricted in avian species, including wild birds and domestic poultry [4], [34]. Previous data showed that some H9N2 isolates from domestic poultry possessed the ability to replicate in mice without prior adaptation, and cause varying levels of lethality [8], [12], [13]. However, such high mortality in mice induced by the H9N2 isolates tested herein is unusual for low pathogenic H9N2 viruses examined in previous studies [8], [12], [13]. It must be emphasized that all six H9N2 viruses in evaluated in this study were low pathogenic for mice as indicated by MLD_50_ (Table 1). That is, the H9N2 viruses caused severe disease and mortality in mice only after exposure to high doses (10^5^–10^7^ EID_50_) of virus. In contrast, high mortality was observed in mice with H5N1 HPAI viral infection at low doses (10^2^–10^4^ EID_50_) [45].

Although we do not know which viral factors are necessary for the high pathogenicity in mice, the efficiency of viral replication certainly is one likely determinant. Our data showed that H9N2 viral infection resulted in high viral titers in mouse lungs, with peak titers occurring on day 5 p.i. In this study, peak viral titers in the lungs corresponded with the onset of respiratory disease.

Several studies have suggested that the presence of Lys at position 627 of PB2 favors the replication of influenza viruses in mammals, and thus it is considered an important host range determinant [42]–[44]. In addition, the amino acid sequence of the connecting peptide of HA is considered another determinant of influenza viral virulence in poultry. That is, a multibasic amino acid HA cleavage site plays an important role in pathogenicity of influenza viruses [35]–[37]. However, in the current study, all six H9N2 viruses had conserved amino residues of Glu-627 in PB2, and possessed the conservative amino acid sequences of PARSSR↓G at the cleavage sites (arrow) between HA1 and HA2, the characteristic of low pathogenic avian influenza viruses in terrestrial poultry [35]–[37], [40]. Therefore, these residues might not contribute to the difference of replication and virulence of our H9N2 viruses in mice. The receptor specificity of the viral HA is reported to be a factor that may limit the generation of human pandemic viruses from avian precursors [8], [25], [26], [38]. A recent study has revealed that the human virus-like receptor specificity, related to the presence of Leu226 in the HA, appears to be associated with transmissibility of H9N2 viruses to mammals (ferrets) [27]. In the present study, four of the six H9N2 isolates contained Leu226 residues at RBS (numbering according to H3 HA) of HA (Table 4), which is typical of human H2 and H3 pandemic strains but not avian influenza viruses [8], [25]–[27], [38], [46]. Whether the presence of Leu226 in the HA of these H9N2 viruses is associated with host adaptation in mice needs to be investigated further.

Another essential question is how H9N2 viruses acquired the ability to replicate in mice. We propose three potential sources of “mammalian adaptation” of H9N2 viruses. One is the potential for terrestrial birds to serve as intermediate hosts. The circulation of H9N2 viruses among domestic poultry for prolonged periods might progressively provide the viral replication property that permits the virus to better adapt to infect mammal (mice). The present study showed that H9N2 viruses with a similar phylogenetic background to that of our isolates had been prevalent in chicken flocks in northern China for more than ten years [13], [15], [17]. Data from recent experimental research provides additional support for this mechanism of mammalian adaptation. It has been shown that laboratory adaptation of a duck H9N2 virus (A/Duck/Hong Kong/702/79) through serial lung passages in quail and chickens generated variant viruses that produced a large-plaque phenotype, showed rapid replication kinetics in tissue culture, and gained the ability to replicate efficiently in mice [47]. Another potential mechanism for mammalian adaptation might involve the use of the pig as an intermediate host in the selection of H9N2 viruses. Pigs are susceptible to infection with a range of avian and human influenza viruses. It has been proposed that pigs can serve as “mixing vessels” for the reassortment of human, avian and swine influenza viruses [48], [49]. In the region of China where the H9N2 viruses tested in this study were isolated, families typically own a small number of pigs and chickens, which are housed in close proximity, especially in farming villages. Under this situation, H9N2 viruses might have gradually acquired the ability to replicate in mammals by means of selection pressure created by possible transmission between pigs and chickens. To date, the repeated H9N2 viral infection of pigs with apparent clinical disease has been reported in China since 1998 [18]–[20]. Furthermore, phylogenetic analysis in the present study revealed that six gene segments (including HA and NA genes) of three swine H9N2 viruses isolated from Shandong province in northern China during 2003–2005 were grouped in the same clusters with the six H9N2 isolates tested (Fig. 1 and Fig. 2). These findings indicate the occurrence of interspecies transmission between chickens and pigs in this region. Finally, mammalian adaptation of the H9N2 viruses could possibly have involved multiple viral genes. Several avian gene constellations have been proposed to promote pathogenicity in mammals. For instance, Qa/HK/G1/97 H9N2 virus, which has six internal genes are closely related to those of H5N1 HPAI viruses, is pathogenic in mice and is able to spread to the brain of infected mice without adaptation [12]. In the current study, we observed that the RNP complex genes in a number of H5N1 HPAI viruses isolated in China after 2001 shared a high identity to those of our H9N2 isolates and also were clustered into the Ck/SH/F/98-like sublineage (Fig. 2). This indicated that reassortment events had occurred between these two subtypes of viruses. Further studies are required to understand whether the gene constellation with Ck/SH/F/98-like RNP complex genes is involved in mammalian adaptation of H9N2 viruses.

The present study indicated that the H9N2 viruses with enhanced pathogenicity to mice have established a stable sublineage in chickens in northern China. This situation could give rise to great uncertainty for the future evolution and ecosystem of H9N2 viruses in China. First, this H9N2 variant might further adapt and possible reassort with other avian influenza viruses, which could cause new outbreaks and promote the introduction of other novel unpredictable reassortants. Thus, future surveillance will determine whether the prevalence of this sublineage of H9N2 viruses in chickens has increased the genetic diversity of circulating avian influenza viruses. Second, this sublineage of H9N2 viruses continue to challenge the species' barrier between birds and mammals and, consequently, may pose a risk for entering into the human population. Therefore, it is imperative that particular attention be paid to the ecology and evolution of avian-originated H9N2 viruses, in order to avert future pandemic in humans.

## Materials and Methods

### Sampling and virus isolation

Lung and oviduct samples were collected from chickens experiencing a severe drop in egg production from different poultry farms in Hebei and Henan provinces in northern China (Table 1). The samples were kept in a sterile plastic bag and stored at −80°C. Each sample was frozen and thawed twice, and the extracted fluid was clarified by low speed centrifugation. The supernatant was filtered through a sterile membrane filter, and a 0.2 ml volume of the filtrates was inoculated into the allantoic cavity of a 10-day-old embryonated SPF hens' egg. Inoculated eggs were incubated at 37°C for 96 h. After inoculation, allantoic fluid (AF) was harvested and tested for hemagglutination activity. The isolates were typed as H9N2 viruses by hemagglutinin inhibition (HI) and neuraminidase inhibition (NI) tests with a panel of reference antisera as previously described [50]. Virus stocks were propagated in allantoic cavities of 10-day-old embryonated SPF chicken eggs at 37°C for 72 h, and stored at −80°C for use in all the experiments described herein. Six representative viruses studied in detail are listed in Table 1. The EID_50_ titers of 6 viruses were determined by serial titration of viruses in 10-day-old embryonated SPF chicken eggs at 37°C. Viral titers were calculated by the method of Reed and Muench as described previously [51]. Because of the potential risk to mammals and poultry, all experiments were conducted under biosafety level 2+ (BSL-2+) laboratory conditions. The study was approved by the Animal Care Committee of China Agricultural University (Beijing, People's Republic of China).

### Genetic and phylogenic analysis

Viral gene sequencing and analysis were carried out as described previously [13]. In brief, viral RNAs were extracted from infectious AF with the TRIZOL LS kit (Invitrogen, Carlsbad, CA, USA) according to the manufacturer's instructions. Reverse transcription (RT)-PCR was performed using primers (primers are available upon request) specific for eight gene segments. PCR products were purified and were cloned into the pMD18-T vector [15], and then DNA templates were sequenced on an ABI377 automated DNA sequencer using reagents from BGI Life Tech Co. Ltd. (Beijing, People's Republic of China) [15]. The nucleotide sequences were compiled, edited and compared by using the DNAMAN program (version 6.0). Phylogenetic analysis was carried out by the neighbor-joining bootstrap analysis (1,000 replicates) using the MEGA program (version 4.1). The nucleotide sequences from this study are available from GenBank under the accession numbers listed in Table 1.

### Chicken experiments

Four-week-old white leghorn SPF chickens (Beijing Laboratory Animal Research Center, Beijing, People's Republic of China) were housed in microisolator cages ventilated under negative pressure with HEPA-filtered air. Pilot experiments indicated that SPF chickens did not exhibit any clinical signs when inoculated intranasally (i.n.) with the doses of less than 10^6^ EID_50_ of H9N2 viruses. Therefore, we evaluated the effect of higher dose of H9N2 viruses in chickens. In this experiment, H9N2-inoculated groups (15 chickens in each group) were inoculated i.n. (100 µl) with infectious AF of each of six H9N2 viruses in phosphate-buffered saline (PBS) at a dose of 10^7^ EID_50_. A control group of 15 birds was inoculated i.n. with an equivalent dilution of noninfectious AF. The birds' behavior, clinical signs, body weight, feed intake and mortality were monitored daily for 21 days. For assessment of viral replication in chicken organs, three chickens from each group were euthanized on days 3, 5, 7, and 14 p.i., and the hearts, livers, spleens, lungs, kidneys, and brains were collected. Clarified homogenates were titrated for virus infectivity in eggs from initial dilutions of tissues (1∶10) as previously described [28]. Virus endpoint titers were expressed as mean log_10_ EID_50_ per 10 milligrams wet tissues ± standard deviation (SD).

### Mouse experiments

To assess the pathogenicity of the six H9N2 viruses in mice, six- to eight-week-old female SPF BALB/c mice (Beijing Laboratory Animal Research Center, Beijing, People's Republic of China) were used for this study. Three experiments were carried out in this study. The first experiment was to determine the MID_50_ and MLD_50_ titers of six isolates. For this purpose, groups of four mice were anesthetized with diethyl ether, and infected i.n. (100 µl) with infectious AF in PBS of each of six H9N2 viruses (10-fold serial dilutions containing 10^1^ to 10^7^ EID_50_ of the virus). Animals were observed for 14 days. MID_50_ and MLD_50_ titers were calculated by the method of Reed and Muench [51]. Lung virus titers were used for the determination of MID_50_. The second experiment was to investigate the clinical signs and mortality of H9N2 infected mice over a 14-day observation period. In this experiment, mice were divided randomly into 25 groups with 14 mice in each group, including 24 infection groups (six virus isolates × four doses) and one control group. H9N2-infected groups were anesthetized with diethyl ether, and then inoculated i.n. (100 µl) with infectious AF of each of six H9N2 viruses diluted in PBS at a dose of 10^4^, 10^5^, 10^6^, or 10^7^ EID_50_, respectively. Mock-infected control animals were inoculated i.n. (100 µl) with an equivalent dilution of noninfectious AF. The animals' general behavior and clinical signs, including food intake, body weight, inactivity, anal temperature (measured with an infrared thermometer) and mortality, were monitored daily for 14 days.

In the third experiment, viral replication was evaluated in mice after H9N2 viral infection. Mice were divided randomly into 19 groups with 30 mice in each. H9N2 infected groups were inoculated i.n. with each of six H9N2 viruses at a dose of 10^5^, 10^6^, or 10^7^ EID_50_, and control group received the noninfectious AF, as described above. Lungs, kidneys, brains, livers, spleens, and hearts of three mice of each group were collected on days 3, 5, 7, and 14 p.i. Left lung lobes were fixed in buffered 10% formalin, embedded in paraffin, and five micrometers-thick sections were stained with hematoxylin-eosin for histopathological evaluation. The right lung lobes and other organs were stored at −80°C for determining viral titers. Virus titration was performed as described in the chicken experiment. Viral titers were expressed as mean log_10_ EID_50_ per 10 milligrams of wet tissues ± SD.
